# Investigation of LAMP Technique in Diagnosis Type of *Plasmodium Species* in Anopheles Mosquitoes :A Fast and Practical Technique to Detect Malaria Pathogens in the Field

**DOI:** 10.4314/ejhs.v31i4.8

**Published:** 2021-07

**Authors:** Hadi Mirahmadi, Nadia Kazemipour, Anis Yazdiani, Ahmad Mehravaran, Hamid Reza Basseri, Leili Mohammadi, Ebrahim Alijani

**Affiliations:** 1 Infectious Disease and Tropical Medicine Research Center, Resistance Tuberculosis Institute, Zahedan University of Medical Sciences, Zahedan, Iran; 2 Department of Parasitology and Mycology, Faculty of Medicine, Zahedan University of Medical Sciences, Zahedan, Iran; 3 Department of Microbiology, Faculty of Basic Sciences, Islamic Azad University, Kerman, Kerman, Iran; 4 Department of Medical Entomology, School of Public Health, Tehran University of Medical Sciences, Tehran, Iran; 5 Clinical Immunology Research Center, Zahedan University of Medical Sciences, Zahedan, Iran

**Keywords:** Malaria, Nested-PCR, LAMP, Anopheles Mosquitoes

## Abstract

**Background:**

Malaria is one of the main parasitic diseases and a major health issue in some countries. This study aims to determine the rate and type of infections of Anopheles mosquitoes with malaria parasites using the molecular LAMP method in the Southeastern Iran.

**Methods:**

In this study, 400 Anopheles mosquitoes were collected by the Zahedan Medical Insecticide Center in Nikshahr City, a high-risk area of malaria transmission in Sistan-Baluchestan Province. The mosquitoes were caught manually (by hand) in domestic (humans and animals), natural, and artificial outdoor places (Shelter pits). After DNA extraction, the LAMP method was used, which was compared with Multiplex Nested-PCR as a standard method.

**Results:**

Out of 400 samples collected from Nikshahr City, 6 samples (1.5%) were infected with Plasmodium vivax. No Plasmodium falciparum or a mix (Plasmodium vivax and Plasmodium falciparum) was detected in this study.

**Conclusions:**

The results of this study indicate that in places with transmission of both species, i.e. Plasmodium vivax and Plasmodium falciparum, detection of malaria parasites by the LAMP method could be very useful in spotting infections in the field. Thus, molecular epidemiological studies could be conducted annually to monitor malaria in endemic regions. The results of this research show that contamination with mosquito malaria vectors is increasing in Nikshahr City, and it seems that more studies will be required to eliminate malaria until 2026.

## Introduction

Malaria is a critical public health issue in tropical and subtropical regions. In humans, four protozoan parasites, including *Plasmodium vivax, P. falciparum, P. ovale*, and *P. malariae* lead to the spread of this mosquito-borne infectious disease. The first treatment line for this disease is diverse and depends on *Plasmodium* species. In other words, proper selection of antimalarial drugs is based on thorough knowledge of pathogens (1–3). Therefore, proper identification of pathogens is important before standard chemotherapy. According to a report released by the World Health Organization in 2016, despite various malaria control and antimalarial programs, 216 million cases of malaria were reported from 91 countries, which exceeded the number in 2016 by about 5 million cases. The annual mortality rate of malaria is around 445,000 individuals worldwide. Malaria deaths declined from 2015 to 2016 in Southeastern Asia but increased in Africa, Eastern Mediterranean, and the Pacific region (4, 5)

Iran is one of the known malaria endemic places in the Eastern Mediterranean region, with its endemic malaria areas being the provinces of Sistan-Baluchestan and Hormozgan, as well as Jiroft and Kahnuj Cities in Kerman Province (6) In Iran, the main goal of the malaria elimination program is to stop local transmission of the disease and to become a malaria-free region in 2026(7). One of the essential strategies in the Global Malaria Control Program is the control of *Anopheles* mosquitoes, which is considered one of the most effective ways to eradicate and reduce malaria transmission around the world. Besides, awareness of the diversity of *Anopheles* species and infection with sporozoites in their salivary glands is one of the most decisive epidemiological steps in planning malaria control and prevention methods appropriate for local conditions (8) Various biochemical, molecular, and immunological methods (9–11), such as isoenzyme electrophoresis, polymerase chain reaction (PCR), and monoclonal antibodies have been used to detect *Plasmodium* in mosquitoes(12, 13). One of the most common detection methods for malaria parasites in *Anopheles* mosquitoes is dissection of salivary glands in female *Anopheles*, captured by stereomicroscopy and light microscopy. However, this method is very cumbersome, time-consuming, and lowly sensitive (12,14).

PCR methods are highly sensitive and specific, capable of detecting 1–10 parasites per microliter of blood, being five times more effective than microscopic detection by Giemsa-stained spreads. In addition to detecting parasite species, PCR methods could detect multiple infections.

Independent PCR detection of all pathogens with corresponding single primers needs more time, effort, and costs in the case of a mixed infection. Multiplex PCR-based approaches are more effective, in which more than one pair of primers are employed in a single response, thereby resulting in different sizes of DNA fragments being amplified simultaneously. In addition, they cut costs, save time, and are effective in detecting mixed infections. Thus, a multiplex nested-PCR method has been employed in the present study for simultaneous detection of *P. vivax* and *P. falciparum*(12). High sensitivity and specificity of this technique indicate its effectiveness in diagnosing asymptomatic malaria cases of a low parasitic burden. Besides, it is a perfect method for detecting malaria in low-transmission areas, which could be used in monitoring drug resistance as well. Furthermore, many samples could be tested simultaneously (12).

Loop-mediated isothermal amplification (LAMP) has made it possible to use a specific, rapid, sensitive, and simple technique for diagnosing a variety of diseases, including vector-borne ones (15). Bst DNA polymerase and a set of four specially designed primers, which identify six separate regions of the target DNA, are employed by LAMP. The target gene could be amplified and detected in an isothermal phase (15–17). Upon accumulation of about 10 ^9^ copies of target DNA in less than one hour, auto-cycling strand displacement DNA synthesis is performed. A series of stem-loop DNA structures of different lengths are included in amplified products (18,19). Simple detection could be performed by visually inspecting turbidity of magnesium pyrophosphate, i.e. a byproduct of DNA synthesis, produced in proportion to the amount of amplified DNA (20, 21). Using a loopamp real-time turbid meter, real-time detection could be performed as well (22). Due to the relatively favorable climate and geographical conditions in the southern and southeastern regions of Iran as well as migration to and from Afghanistan and Pakistan, malaria is a major public health issue in Iran. Sistan-Baluchestan Province has a long border with Pakistan, across which the largest number of malaria cases is observed (23). This study aims to examine the rate and type of *Anopheles* mosquito infections with sporozoites by Multiplex Nested-PCR and LAMP, which is performed in Nikshahr City of Sistan-Baluchestan Province.

## Methods

**Figure 1:**
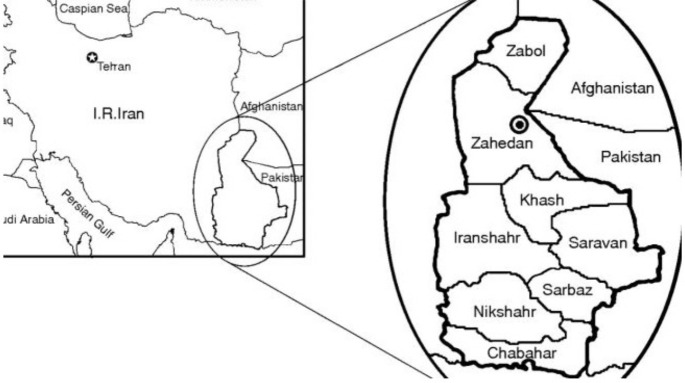
Geographical locations where this study was carried out

**Sample preparation**: This descriptive cross-sectional study was conducted in the time period from March 2018 to July 2019 in Nikshahr City, a high-risk area of malaria transmission in Sistan-Baluchestan province. Populations surveyed in the city included Bennett, Fanuj, Lashar, Ahuran, and Markazi sampled from selected villages every 15 days, in which blood-fed *Anopheles* mosquitoes were captured (Figure 1). The *Anopheles* mosquitoes were caught manually (by hand) in domestic (humans and animals), natural, and artificial outdoor sites (Shelter pits), with suction tubes or aspirators utilized. All aspirating specimens were killed by a small chloroform-impregnated cotton pad placed in plastic cups. Just after the collection task, the samples were placed in 70% alcohol-containing tubes and transferred to the relevant health center. Using a high magnification entomology loop, the blood-fed *Anopheles* mosquitoes were separated and transferred into 1.5 ml tubes. Next, each sample was assigned a three-letter code and then stored in the tubes at -20 °C until DNA extraction.

**Multiplex Nested-PCR Method**: DNA extraction was performed by the column method through employing a DNA extraction kit (Produced by the DynaBio Blood/Tissue Genomic DNA Extraction Kit). Multiplex Nested-PCR was performed to determine species of *Plasmodium* via employing designed primers (Table 1). A pair of oligonucleotide primers (rPLU6 and rPLU5) was employed for the PCR-1 reaction in this study. In this reaction, primers, including rFAL.1, rFAL.2, rVIV.1, and rVIV.2 were used to simultaneously determine *Plasmodium* species (13, 24). The first PCR product was diluted at a 1:100 ratio with distilled water as a template in the second PCR. The amplified products were electrophoresed on 2% agarose gels in Tris-borate-EDTA for *P. vivax* and *P. falciparum* and then by the ultraviolet Trans illuminator stained with a red gel for visual detection.

**Table 1:** Sequences of the primers used in the study for representation of the *Plasmodium* ssrRNA genes and multiplex/nested PCR protocol

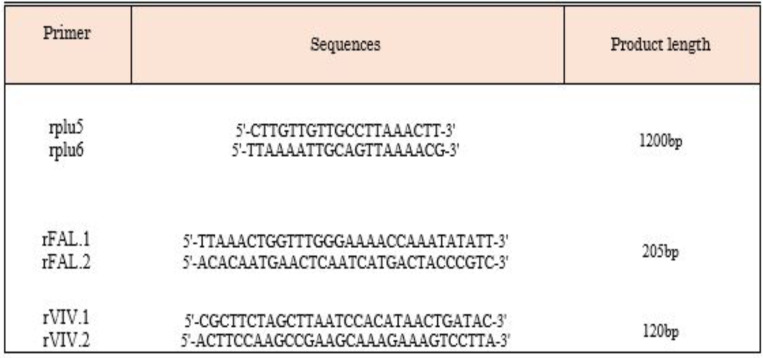

The PCR steps included denaturation of the DNA molecule at 95 °C for 5 min and at 94 °C for 30 s, with 25 cycles for the first step and 30 cycles for the Nested-PCR, annealing at 58 °C for 1 min, extension at 72 °C for 60 s for the first step and 30 seconds for the Nested-PCR, final extension at 72 °C for 10 min for the first step and 5 min for the Nested-PCR, and finally, completion of the reaction and a reduction in the temperature to 20 °C (22,25,26).

**LAMP reaction**: Table 2 provides information about the LAMP primer for amplifying gene coding for 18S rRNA specific *Plasmodium* genus (27, 28). The LAMP reaction mixture (25 µL) included 1 µl of template DNA (100 ng/µL), 40 pmol of each of the FIP (Forward Internal) and BIP (Backward Internal) primers, 20 pmol of primer LB (Loop Backward), 5 pmol of each of the F3 (Forward 3) and B3 (Backward 3) primers, 8 U Bst2 DNA polymerase (New England Biolabs, Ipswich, MA, USA), 2 × reaction buffer of 40 mM Tris-HCl (pH 8.8), 20 mMKCl, 20 mM (NH_4_)_2_SO_4_, 16 mM MgSO_4_, 0.2% Tween 20, 0.8 M betaine (Sigma-Aldrich), as well as 1.4 mM deoxynucleoside triphosphates (dNTP) according to Table 3.

**Table 2:** Sequences of the LAMP primer used in the study for representation of the 18S rRNA specific *Plasmodium* genus

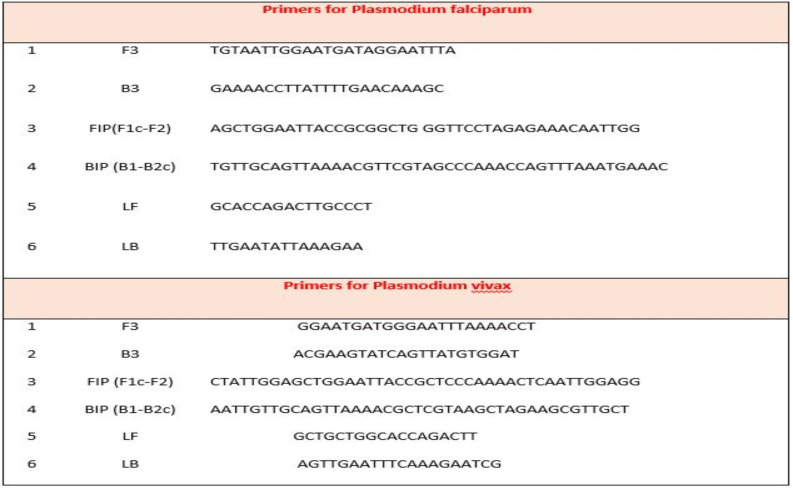

**Table 3:** LAMP reagents for both *Plasmodium* species

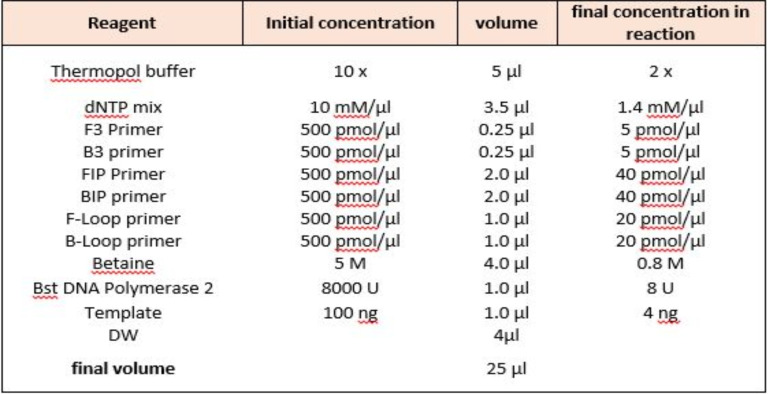

The amount of reagents was computed based on the total number of the samples, which was provided as a master mix. Next, 24 µl of the mixture was poured separately into the new microtubes, and 1 µl of each sample's DNA was added next to each microtube. The reaction mixture was incubated in a water bath for 60 min at 60 °C and inactivated for 2 min at 80 °C. SYBR Green I (Invitrogen Carlsbad, CA, USA) was added to the reaction microtubes, and the resulting amplicons were spotted by visual observation. By triggering the reactions for 30, 45, 60, and 75 min at 60–64 °C in duplicate, optimal time and temperature conditions for the LAMP assay were defined. Besides, a LAMP reaction would be considered positive for *Plasmodium spp* if naked eyes detected an apparent rise in turbidity as opposed to the negative control. Two researchers assessed the results blindly.

**Statistical analysis**: Diagnostic performances of the LAMP method were evaluated using multiplex/nested-PCR as the standard method. SPSS 16.0 (SPSS Inc., Headquarters USA) was employed to perform the analyses. To establish consistency of the results among the diagnostic tools, the Kappa value was defined.

## Results

**Figure 2:**
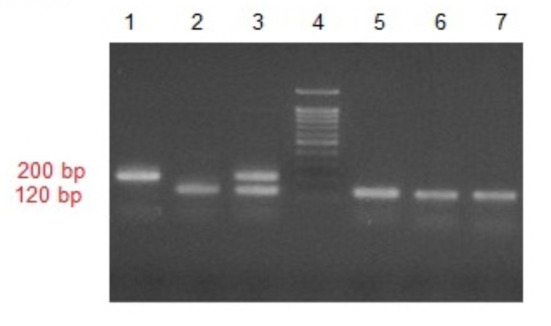
Electrophoresis of the ssrRNA gene multiplex/nested PCR products on a 2% agarose gel, Lane1: Positive control of Plasmodium falciparum (205 bp), Lane2: Positive control of Plasmodium viv(120 bp), Lane3: Positive control of mixed infections, Lane4: Ladder 100 bp, Lanes 5,6,7: P. vivax

**Multiplex Nested-PCR**: In the present study, the samples (400 blood-fed *Anopheles* mosquitoes) were collected within the time period from March 2018 to July 2019 in a highrisk region of malaria transmission in Sistan-Baluchestan Province, Nikshahr City. Out of 400 samples, 6 (1.5%) samples were infected with *Plasmodium vivax* via Multiplex Nested-PCR. In this method, no infection with *Plasmodium falciparum* and the mix (*Plasmodium vivax* and *Plasmodium falciparum*) was observed (Figure 2).

**Figure 3:**
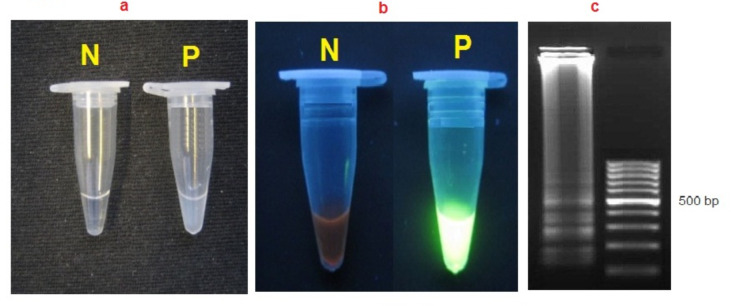
Visualization of LAMP products with SYBR Green I a under natural light, b under UV light and c agarose gel electrophoresis. N no template control, P positive control

**LAMP reaction**: Upon addition of SYBR Green I to the reaction microtubes, the positive LAMP reaction was visually detectable; in the LAMP technique, the positive reaction turned green, and the negative one remained orange (Figs. 3a and 3b). Besides, the positive LAMP reaction produced a typical ladder-like pattern of multiple bands of various sizes until well loaded on a 2% agarose gel stained with a red gel (Figure 3c).

When the LAMP reaction time and temperature were kept fixed for 90 min at 60 °C for the 18SSU rRNA genomic target, the optimum results were achieved. Similar to the Multiplex Nested-PCR method, 8 (2%) out of 400 samples were infected with *Plasmodium vivax* by the LAMP technique. Like the previous method, no infection with *Plasmodium falciparum* or the mix (*Plasmodium vivax* and *Plasmodium falciparum*) was observed. According to Kappa measure of agreement, both methods were completely consistent with each other (K=1).

## Discussion

Malaria is still a leading health issue in the southeastern regions of Iran, especially in Sistan-Baluchestan Province. In addition, it is effective in preventing economic and social growth in these deprived regions (29). In the present study, two molecular methods, including Multiplex Nested-PCR and LAMP, were used for the first time in Iran to detect malaria infections in vector mosquitoes. The mentioned two methods were highly sensitive, identified the genus and species of the parasites, and could detect mix infections. The current study was conducted in Nikshahr City, one of the high-risk areas of malaria transmission in Sistan-Baluchistan Province. In this study, a total of 400 blood-fed female *Anopheles* mosquitoes were captured. The results of this study showed that the dominant species of *Anopheles* mosquitoes in the studied area was *A. culicifacies* (83.33%).

*Plasmodium vivax* is the most prevalent malaria species in Iran, which is confirmed by contamination of 1.5% of the mosquitoes in Nikshahr City, being in line with findings of other studies (30). A similar study was conducted in Chabahar and Iranshahr by Oshaghi et al (2004), in which *Plasmodium vivax* was found to be the predominant species in the captured blood-fed female mosquitoes (31).

Assmar et al (2005) conducted a study in endemic regions of Iran using PCR, and the results of which showed that *Plasmodium vivax* was the dominant parasite in the studied regions (32). Kalantari et al (2017) conducted a study in Fars Province, in which PCR identified a total of 512 female *Anopheles* mosquitoes. The results showed that *Anopheles dthali* (71.38%) was the dominant species in the mentioned region, yet no *Plasmodium* parasite infection was reported by mosquito PCR. However, in the present study, infections (1.66%) were reported in *Anopheles* mosquitoes. Sistan-Baluchestan Province is one of the endemic regions of malaria, with the difference between its endemic status and that of Fars province having caused the present study to report *Plasmodium* parasitic infections in *A. stephensi* (33).

In a similar study in India, 523 Anopheles mosquitoes were studied to detect infection with *Plasmodium*. The results of this study showed that *Anopheles annularis* was the predominant species, yet no sporozoites were observed after salivary gland separation and microscopic examination. However, 7 *Anopheles annularis* mosquitoes (3.4%) were infected with *Plasmodium falciparum* by PCR (34). In another study in the Western India, Malaria infection in *Anopheles* was investigated by the Nested-PCR method, according to which, 2.8% of *Anopheles subpictus* were found to be contaminated with a mix of *Plasmodium falciparum* and *vivax* (35).

Many factors influence prevalence of *Plasmodium* species in different regions of the world. Climatic and ecological differences could be very effective in this regard. Determining the composition of *Anopheles* species and the amount of sporozoites in their salivary glands by reliable and sensitive methods is crucial for malaria prevention plans (8).

This study shows that the Multiplex Nested-PCR method could be effectively used for a large number of samples. This procedure does not require to dissect salivary glands, yet it can even examine dried and old specimens. Therefore, there is no need for equipping laboratories and dispatching personnel to malaria endemic regions because samples collected in these regions could be tested at central laboratories. On the other hand, the LAMP method employed for the first time in Iran to detect malaria in mosquitoes is simple, sensitive, and practical, which needs no expensive facilities and equipment. Besides, it could be used with a thermos of boiling water to easily detect contaminants in the field without wasting any time.

Afghanistan and Pakistan are recognized as the most dangerous countries for malaria transmission. Accordingly, since a large number of job seekers in Sistan-Baluchestan Province, especially in NikShahr City, are from these two countries, Iran remains on the list of malariaendemic countries (3, 30, 36). In a study in Konarak County, the reason for focal changes of malaria and its increasing incidence in 2008 was found to be presence of aliens (37). In another research, Raisi et al. reported that most non-indigenous malaria cases in Iran's Sistan-Baluchestan Province were emanated from bordering cities of Baluchestan State of Pakistan (38). In addition, Poudat et al. investigated factors affecting a malaria reduction in Bandar Abbas City and concluded that the natives of that city were always at the risk of malaria epidemics due to the presence of immigrants and movement of aliens (39). Therefore, the importance of migration as a major source of the crisis and a major challenge in controlling malaria should be considered.

Epidemiological investigations show that malaria in Iran, unlike that in Africa and other countries, is subject to climatic conditions and on the verge of eradication (25, 26). This implies that this unstable state could contribute to the concern that increased rainfalls in high-risk regions could be associated with the higher prevalence of the disease (40). Climatic conditions, socioeconomic and cultural factors, quantity and quality of malaria control programs, common borders with Afghanistan and Pakistan, and issues related to Afghan and Pakistani immigrants are causative factors in the annual incidence of the disease in Sistan-Baluchestan Province (36). For the reasons mentioned above and due to the fact that Sistan-Baluchestan province is in the course of eliminating malaria, one of the most effective ways to monitor the abovementioned program is infection observation in *Anopheles* mosquitos. The results of this study, as against past studies, showed that malaria vector mosquito infection in Nikshahr City was increasing. Furthermore, it seems that elimination of malaria until 2026 requires further research.

The results show that in places with transmission of both species, i.e. *Plasmodium vivax* and *Plasmodium falciparum*, multiplex nested-PCR could be very effective in diagnosing malaria parasites for detection of mixed cases of the disease in both humans and vector mosquitoes. On the other hand, according to the results, the molecular LAMP method is very useful in detecting infections in the field due to its high sensitivity, short test time, and the need for inexpensive equipment. According to the Kappa's measure of agreement, both methods are fully consistent with each other (K=1). Thus, molecular epidemiological studies must be conducted annually to monitor this disease in endemic regions. According to the studies mentioned above and the present one, malaria vector mosquito infection is increasing in Nikshahr City. Given that Sistan-Baluchestan Province is in the course of eliminating malaria, failure to control this disease could lead to an epidemic. Accordingly, continuous and substantial control measures are required to be implemented in all regions of the country. Furthermore, all border entry points need to be controlled, with illegal immigrants required to be prevented from entering the country.
